# Molecular Characterization and Expression Analysis of ATP-Gated P2X7 Receptor Involved in Japanese Flounder (*Paralichthys olivaceus*) Innate Immune Response

**DOI:** 10.1371/journal.pone.0096625

**Published:** 2014-05-05

**Authors:** Shuo Li, Xuejing Li, Claudio Coddou, Xuyun Geng, Junli Wei, Jinsheng Sun

**Affiliations:** 1 Tianjin Key Laboratory of Animal and Plant Resistance, College of Life Sciences, Tianjin Normal University, Tianjin, China; 2 Department of Biomedical Sciences, Faculty of Medicine, Universidad Catolica del Norte, Coquimbo, Chile; 3 Tianjin Center for Control and Prevention of Aquatic Animal Infectious Disease, Tianjin, China; University Paris Sud, France

## Abstract

ATP-gated P2X7 receptor (P2RX7) channel is a key component for purinergic signaling and plays important roles in the innate immune response in mammals. However, the expression, molecular properties and immune significances of P2RX7 in lower vertebrates are still very limited. Here we identified and characterized a novel bony fish *P2RX7* homologue cDNA, termed *poP2RX7*, in Japanese flounder (*Paralichthys olivaceus*). PoP2RX7 protein shares about 60–88% sequence similarity and 45–78% sequence identity with known vertebrate P2RX7 proteins. Phylogenetic analysis placed poP2RX7 and other P2RX7 proteins within their own cluster apart from other P2RX members. While the functional poP2RX7 channel shares structural features in common with known P2RX7 homologs, electrophysiological studies revealed that BzATP, the more potent agonist for known mammalian and fish P2RX7s, shows similar potency to ATP in poP2RX7 activation. *poP2RX7* mRNA constitutively expressed in all examined tissues from unstimulated healthy Japanese flounder with dominant expression in hepatopancreas and the lowest expression in head kidney, trunk kidney, spleen and gill. *poP2RX7* mRNA expression, however, was significantly induced in Japanese flounder head kidney primary cells by Poly(I:C) and bacterial endotoxin LPS stimulations. *In vivo* experiments further revealed that *poP2RX7* gene expression was substantially up-regulated by immune challenge with infectious bacteria *Edwardsiella tarda* and *Vibrio anguillarum*. Moreover, activation of poP2RX7 results in an increased gene expression of multifunctional cytokines *IL-1β* and *IL-6* in the head kidney primary cells. Collectively, we identified and characterized a novel fish P2RX7 homolog which is engaged in Japanese flounder innate immune response probably through modulation of pro-inflammatory cytokines expression.

## Introduction

The purinergic P2X receptors (P2RXs) composed of seven members in vertebrates, termed P2RX1-7, are a family of ligand-gated membrane ion channels that open in response to the binding of extracellular ATP [1]. P2RX subunits exhibit overall similar topological structures: two membrane-spanning domains, separated by a large extracellular loop with both N and C termini in cytosol [2] and these subunits may assemble as homo- or hetero-trimers to form functional receptors. Compared with other P2RXs, however, P2RX7 has a unique long C terminus with an extra 200 amino acid residues containing multiple protein and lipid interaction motifs, including a conserved lipopolysaccharide (LPS) binding domain [3], a tumor necrosis factor (TNF) receptor 1 homology domain [4], and a cysteine-rich 18-amino acid segment, which are implicated in regulating receptor cellular localization, protein–protein interactions, post-translational modification [5], and pro-inflammatory effects [6]. In addition, functional P2RX7 was evidenced to assemble as a homo-trimer with three same subunits [7], [8]. Moreover, P2RX7 has a ubiquitous distribution [9] but expresses in greatest amounts in macrophages, dendritic cells, monocytes, natural killer cells, B-lymphocytes, T-lymphocytes and erythrocytes [10]. Furthermore, P2RX7 requires at least a 100-fold higher ATP concentration for activation than is required for other P2XRs, and removal of divalent cations can increase its agonist potency [4]. In line with the observation that P2RX7 predominantly expresses in the immune cells/organs, activated P2RX7 by extracellular ATP following tissue injury or infection has been evidenced to play a central role in mammalian innate immune responses through the secretion of pro-inflammatory cytokines IL-18 and IL-1β [4], induction of apoptosis [11], generation of reactive oxygen and nitrogen intermediates [12] and stimulation of phagosome–lysosome fusion [13]. Consequently, P2RX7 has received much more research interests than other P2RXs because of these distinctive properties.

*P2RX7* cDNAs have been found in human, mice, dog and several other vertebrate species since it was cloned from rat macrophages by Surprenant et al. in 1996 [14]–[17]. In teleost, *P2RX7* orthologues have been identified from zebrafish [18], seabream [19] and ayu [20]. Available literature has documented that seabream P2RX7 exhibits different agonist (ATP/BzATP)-evoked pharmacological responses from mammalian and zebrafish P2RX7s [19], suggesting species differences of P2RX7 in agonist/antagonist action may exist in teleost. Previous studies also indicate that P2RX7 may play a vital role in fish innate immunity [19], [20]. Compared with the intensive studies in mammals, however, the channel properties and biological significances of P2RX7 in fish are still limited. Given the great species diversity and increasing economic importance, more details about fish P2RX7s are therefore needed to understand the biological significances of this receptor in fish. For this purpose, here we identified and characterized a new bony fish *P2RX7* homologue cDNA (namely *poP2RX7*) from Japanese flounder *Paralichthys olivaceus*, which is one of the most important mariculture fish species for human consumption and contributes a major part in mariculture industry in China. We also investigated the *poP2RX7* gene expression profile in response to different immunological challenges and its potential role in regulating the gene expression of multifunctional cytokines *IL-1β* and *IL-6*. Our experiments indicate that a novel *P2RX7* homolog, *poP2RX7*, implicated in Japanese flounder innate immune response has been identified and characterized.

## Materials and Methods

### Ethics statement

All experiments were conducted in accordance with the NIH guidelines for the care and use of experimental animals and these studies were specifically approved by the animal care and use committees of Tianjin Normal University and Universidad Catolica del Norte.

### Animals and maintenance

Japanese flounders (*P. olivaceus*) were purchased from a local farm in Dagang, Tianjin, China, transported to the laboratory and maintained in aerated running seawater aquaria at 17°C for two weeks before experiments. Animals were fed with a commercial pellet diet twice at a ratio of 2% body weight per day and only healthy animals were selected in experiments. For tissue collection, *P. olivaceus* was euthanized with 0.25 g/L tricaine methane sulfonate (Sigma) and the individual tissue was then dissected aseptically.

### Cloning of Japanese flounder *poP2RX7* cDNA

Total RNA from head kidney of *P. olivaceus* was purified by the TRIzol reagent (Invitrogen) and treated with deoxyribonuclease I, amplification grade (Invitrogen) to remove genomic contamination. The integrity of RNA was assessed by electrophoresis on a 1.2% formaldehyde-denatured agarose gel stained with ethidium bromide. The quantity of RNA was determined by measuring OD_260_ with a NanoDrop 2000 UV/Vis spectrophotometer (Thermo Fisher Scientific). SuperScript III RNase H^−^ reverse transcriptase (Invitrogen) was used to synthesize first-strand cDNA with an oligodeoxythymidine adaptor primer (5′-TCGAATTCGGATCCGAGCTCT_17_V-3′) from 5 µg of total RNA at 50°C for 50 min according to the manufacturer's instructions.

For cloning of *poP2RX7* gene, degenerate primer pair F1/R1 (Table 1) was designed based on the conserved regions of P2RX7 amino acid sequences from different vertebrate species and PCR was performed. PCR products were separated by a 1.2% agarose gel containing 0.5 g/µl ethidium bromide and visualized under UV light. A distinct PCR product with expected size (299 bp) was excised and purified with the Wizard PCR Prep DNA Purification System (Promega). The purified PCR products were then subcloned into pGEM-T Easy vector (Promega) and sequenced. Identity blast search against GenBank database indicates that this PCR amplicon represents a new homologue of *P2RX7* gene family. This partial Japanese flounder *P2RX7* nucleotide sequence was used to design gene-specific primers in order to obtain the 5′- and 3′-untranslated region (UTR) of *poP2RX7* cDNA.

**Table 1:** Sequence of primers used in this study.

Primer name	Sequence (5′→3′)
F1	GTTWCWGCKTGGTGYCCWATTG
R1	TYCAGGTYRCAGTCCCAKTTGA
F2	TCCCTGCGTTCAACTTCATCC
R2	TCGAATTCGGATCCGAGCTC
F3	ACGACTCGCTGTGTCCCATCTTC
GSP1	AGTCCAGAAACAAGGTGGTCAGAGCGTAGT
UMP-L	CTAATACGACTCACTATAGGGCAAGCAGTGGTATCAACGCAGAGT
UMP-S	CTAATACGACTCACTATAGGGC
GSP2	CAGCGTCTTGTTGCTCTCTTTCTCATCC
NUP	AAGCAGTGGTATCAACGCAGAGT
F4	AGGGGAGCCAAGGAATGAGTC
R3	CGGCACTGACCAAGCAGATCCAA
F5	CCGAAATACTCCTTCAGACGC
R4	CGCCTGTCCGAACACCAT
F6	AGGTTCCGTTGTCCCG
R5	TGGTTCCTCCAGATAGCAC
F7	CCTGTCGTTCTGGGCATCAA
R6	CACCCCGCTGTCCTGCTT
F8	CAGCTGCTGCAAGACATGGA
R7	GATGTTGTGCGCCGTCATC
GFPF	CCGCTCGAGATGCCGTGCTGCCGTG
GFPR	CCGGAATTCTCACGCATGTCCGTCAGCGCA

The full-length sequence of *poP2RX7* cDNA was obtained by rapid amplification of cDNA ends (RACE) strategy. The 3′-terminal of *poP2RX7* cDNA end was amplified by two rounds of nested PCR. To increase the specificity of amplification, the initial round of PCR was conducted with a gene-specific forward primer F2 (Table 1) designed based on the sequence obtained above and a reverse adaptor primer R2 (Table 1), using the first-strand cDNA synthesized above as the template. The first round PCR products were then diluted 100 times and used as the templates and the second round of PCR was performed again using primer pair F3/R2 (Table 1). The 5′-RACE was performed using a SMART RACE amplification kit (Clontech) by two rounds of nested PCR. The first-strand cDNA was synthesized according to the manufacturer's protocol, and two reverse gene-specific primers GSP1 and GSP2 (Table 1) were used in the nested PCR. The first round of PCR was performed with a forward primer UPM (a mixture of primers UPM-L and UPM-S, Table1) and a reverse gene specific primer GSP1, followed by a nested amplification with primers NUP/GSP2 (Table 1) in a MyCycler gradient thermocycler (Bio-Rad) under the following conditions: 5 cycles of 50 s at 94°C; 50 s at 69°C; 1 min 30 s at 72°C; and 30 cycles of 50 s at 94°C; 50 s at 61°C; 1 min 30 s at 72°C, followed by a final extension of 10 min at 72°C. The fragment obtained was cloned, sequenced and used to design the specific primer F4 (Table 1) in the 5′-UTR. This primer was combined with primer R3 (Table 1) corresponding to the 3′-UTR of *poP2RX7* mRNA, in a confirmation PCR aiming to obtain the complete sequence of *poP2RX7* cDNA. The confirmation PCR products were cloned and completely sequenced on both strands. Computer analyses revealed that they matched exactly with the sequence derived from the results of RT-PCR, 5′- and 3′-RACEs.

### DNA and protein sequence analyses

All nucleotide sequences were blast against GenBank database using BlastX algorithm at the National Center for Biotechnology Information (http://www.ncbi.nlm.nih.gov/blast) to identify their coding proteins. The nucleotide and derived protein sequences of poP2RX7 were compared with other known P2RX7 sequences currently available at GenBank database (www.ncbi.nlm.nih.gov). Multiple sequence alignments were performed using ClustalW multiple alignment program at the European Bioinformatics Institute (http://www.ebi.ac.uk/clustalw/) [21]. The conserved protein domain was predicted through the web site (http://www.ncbi.nlm.nih.gov/Structure/cdd/wrpsb.cgi). Phylogenetic and molecular evolutionary analysis was conducted using MEGA (Molecular Evolutionary Genetics Analysis) software version 5.1 [22]. The phylogenetic tree was constructed based on the amino acid sequence alignments and tested for reliability using 1000 bootstrap replications.

### Tissue distribution analysis of *poP2RX7* mRNA in healthy *P. olivaceus*

Quantitative real-time PCR was employed to investigate *poP2RX7* gene expression in different tissues of healthy *P. olivaceus*. Tissues including brain, blood, gill, head kidney, trunk kidney, heart, hepatopancreas, skin, gonad, muscle, intestine and spleen from five individual healthy animals (average 500±20 g) were dissected and collected. Each tissue was equally pooled to minimize individual variability and stored in RNAlater solution (Ambion) for later use. Total RNA was extracted as described above and aliquots (1 µg) of total RNA from different tissues were transcribed into cDNAs in 20 µl reaction mixtures using SuperScript III ribonuclease H^-^ reverse transcriptase (Invitrogen). To confirm that samples were not contaminated with genomic DNA, negative control reactions for RT-PCR were performed in absence of cDNA template (without transcription) and no PCR products were amplified (data not shown).

### Cell culture and DNA construct

Japanese flounder head kidney primary cells were prepared as described by Li et al [23]. Briefly, head kidneys were dissected and gently passed through a 40 µm sterile cell strainer (BD Biosciences) with a glass homogenizer and rinsed twice with DMEM-F12 medium (Invitrogen) containing 100 U/ml penicillin-streptomycin and 10 U/ml heparin (Sigma). Primary head kidney cells were then cultured at 21°C in a 24-well plate with DMEM-F12 medium supplemented with 10% FBS and 1% penicillin–streptomycin liquid.

The coding region of *poP2RX7* gene was amplified by PCR with PfuUltra II fusion HS DNA polymerase (Stratagene) and primer pair GFPF and GFPR (Table 1) using the full-length *poP2RX7* cDNA plasmid as the template. The PCR products were purified, digested with *EcoR*I and *Xho*I (Fermentas), and cloned into the expression vector pIRES2-EGFP for electrophysiology recording purpose. The correct sequence of the recombinant plasmid was confirmed by DNA sequencing. Large-scale plasmid DNAs were prepared using a QIAfilter Plasmid Maxi kit (Qiagen).

### Electrophysiology recordings

A segment of the ovary was surgically removed under anaesthesia from females of the African frog, *Xenopus laevis*. All procedures of animal handling were done following the NIH protocols. Oocytes were manually defolliculated and incubated 30 min with type III collagenase as previously described [24]. The pIRES-EGFP/poP2RX7 plasmid was generated as described above. Oocytes were injected intranuclearly with 4 ng Japanese flounder or rat *P2RX7* cDNA. After a 12–48 h incubation in Barth's solution (in mM) [88 NaCl, 1 KCl, 2.4 NaHCO_3_, 10 HEPES, 0.82 MgSO_4_, 0.33 Ca(NO_3_)_2_, 0.91 CaCl_2_; pH 7.5] supplemented with 10 U/L penicillin/10 mg streptomycin and 2 mM pyruvate, oocytes were clamped at −70 mV using the two-electrode voltage-clamp configuration with an OC-725C clamper (Warner Instruments Corp., Hamden, CT, USA). ATP-gated currents were recorded following regular ATP or BzATP applications. Washout periods ranged from 5 to 15 min depending on agonist concentration. The recordings were performed either in Barth's or in a low-divalent (LD) containing media (in mM) [91 NaCl, 1 KCl, 0.5 CaCl_2_, 0.1 MgCl_2_, 10 HEPES; pH 7.5]. To determine changes in receptor permeability oocytes expressing poP2RX7 or rP2RX7 were bathed in NMDG^+^ media that contained (in mM) [92 NMDG^+^, 10 HEPES, 0.2 niflumic acid; pH 7.5]. I-V relations were used to evaluate changes in reversal potential and were obtained by 210 consecutive voltage ramps from −100 to +40 mV (duration of each ramp 1 S), delivered during 3 min of ATP application. Non-injected oocytes did not evoke currents when exogenous ATP or BzATP was applied. ATP, BzATP and antagonists were dissolved in Barth's or LD media and perfused using a peristaltic pump operating at a constant flow of 2 ml/min. Concentration-response curves were performed by applying for 20 sec, increasing concentrations of the nucleotide ranging between 3–10000 µM. Curves were normalized against the concentration of ATP that evoked the maximal response. For antagonist experiments, the compound were pre-applied for 2 min and then co-applied with the agonist for 30 sec. The currents obtained under these conditions were compared and normalized against the control responses. The ATP or BzATP median effective concentration (EC_50_) was interpolated from each concentration-response curve using the Prism 5 software (GraphPad). Each protocol was performed in at least two separate batches of oocytes from different frogs and each experiment was repeated at least in four separate oocytes. Statistical differences were analyzed by Mann-Whitney test.

### *poP2RX7* gene expression in response to LPS and Poly(I:C) stimulations in *P. olivaceus* primary head kidney cells

Japanese flounder head kidney primary cells (2.5×10^6^/well) were cultured at 21°C in a 24-well plate (Thermal Scientific) overnight, and then stimulated with LPS or Poly(I:C) (final concentration 25 µg/ml, Sigma-Aldrich). Samples were taken after 0, 2, 4, 6, 8, 12 and 24 h of LPS or Poly(I:C) administration. RNA isolation and cDNA synthesis was performed as described above. The changes of *poP2RX7* gene expression in response to LPS and Poly(I:C) stimulations were investigated by means of quantitative real-time RT-PCR.

### *poP2RX7* gene expression during bacterial infections

Bacteria *Edwardsiella tarda* and *Vibrio anguillarum* isolated from sick *P. olivaceus* were stocked in the lab and cultured from a single colony in marine Luria-Bertani medium overnight. For challenge experiments, healthy Japanese flounder (average weight 20 g) was injected intraperitoneally with 20 µl of *E. tarda* (1×10^6^ CFU per fish) or *V. anguillarum* (1.5×10^7^ CFU per fish) suspended in sterilized physiological saline. Control animals were injected with physiological saline only. Representative tissues involved in fish innate immune response including spleen, head kidney and gill were selected to determine the effects of bacterial infection on *poP2RX7* gene expression *in vivo*. Five individual animals were sacrificed at 0, 4, 8, 12, 24, 36 and 48 h after injection at each time point, and individual tissues were dissected and collected. Each tissue was pooled and RNA was prepared and transcribed as mentioned above. Quantitative real-time PCR was performed to determine the gene expression pattern of *poP2RX7* in response to the bacterial infections.

### PoP2RX7-mediated cytokine gene expression

To examine the involvement of PoP2RX7 in ATP-evoked cytokine gene expression, overnight cultured Japanese flounder head kidney primary cells (1.0×10^7^/well) were pre-incubated with or without selective P2RX7 inhibitors (BBG or A-740003, Sigma) for 2 h and then treated with ATP or BzATP for 30 min to activate poP2RX7. The treated cells were finally incubated with normal culture medium for 2 h and used for RNA isolation. To test the involvement of poP2RX7 in LPS-induced *IL-1β* gene expression, the primary cells were pre-treated with or without P2RX7 inhibitor A-740003 for 2 h and then stimulated with 20 µg/ml LPS for 3 h in the presence or absence of the P2RX7 inhibitor. RNA was then purified using RNeasy mini kit (Qiagen) and transcribed into cDNAs as described above. The gene expression changes of cytokines *IL-1β* and *IL-6* were determined by quantitative real-time PCR.

### Quantitative real-time PCR

Quantitative real-time PCR (qRT-PCR) was performed on a MyiQ^TM^2 Two-Color Real-Time PCR Detection System (Bio-Rad) using SYBR PrimeScript Ex Taq II kit (TaKaRa) according to the manufacturer's instructions. *β–actin* was served as an internal reference gene. The prime pairs used for qRT-PCR detection of *poP2RX7*, *β-actin*, *IL-1β* and *IL-6* are F5/R4, F6/R5, F7/R6 and F8/R7, respectively (Table 1). qRT-PCR was performed using an initial denaturation at 95°C for 30 s, 40 cycles at 95°C for 5 s, 60°C for 30 s followed by dissociation curve analyses (55°C to 95°C: increment 0.5°C for 5 s). Relative expression levels of the target genes in experimental group versus those in control group were determined with the comparative 2^−△△Ct^ quantification method [25]. Data are presented as means ± standard deviation from triplicate experiments. Statistical analysis was performed with Student's *t*-test for the comparison between two groups. Multiple group comparison was conducted by one-way ANOVA followed by Duncan's analysis. Differences were considered significant at *p*<0.05.

## Results

### Isolation of P. olivaceus poP2RX7 cDNA

Using RT-PCR and RACE strategy, a *P2RX7* homologue cDNA, *poP2RX7*, was amplified from the head kidney tissue of *P. olivaceus*. As shown in Fig. S1, the complete *poP2RX7* cDNA sequence including a poly(A) tail derived from the mRNA of Japanese flounder is 2058 bp. It contains a 110 bp 5′-untranslated sequence, an open reading frame consisting of 1743 bp, and a 205 bp 3′-untranslated sequence with a poly(A) tail. A putative polyadenylation signals (AATAAA) was localized at the nucleotide position 2022, which is 9 nucleotides upstream of the poly(A) tail. The cDNA sequence has been deposited in GenBank database under accession number KC748421.

### Sequence analysis of poP2RX7 protein

**Figure 1:**
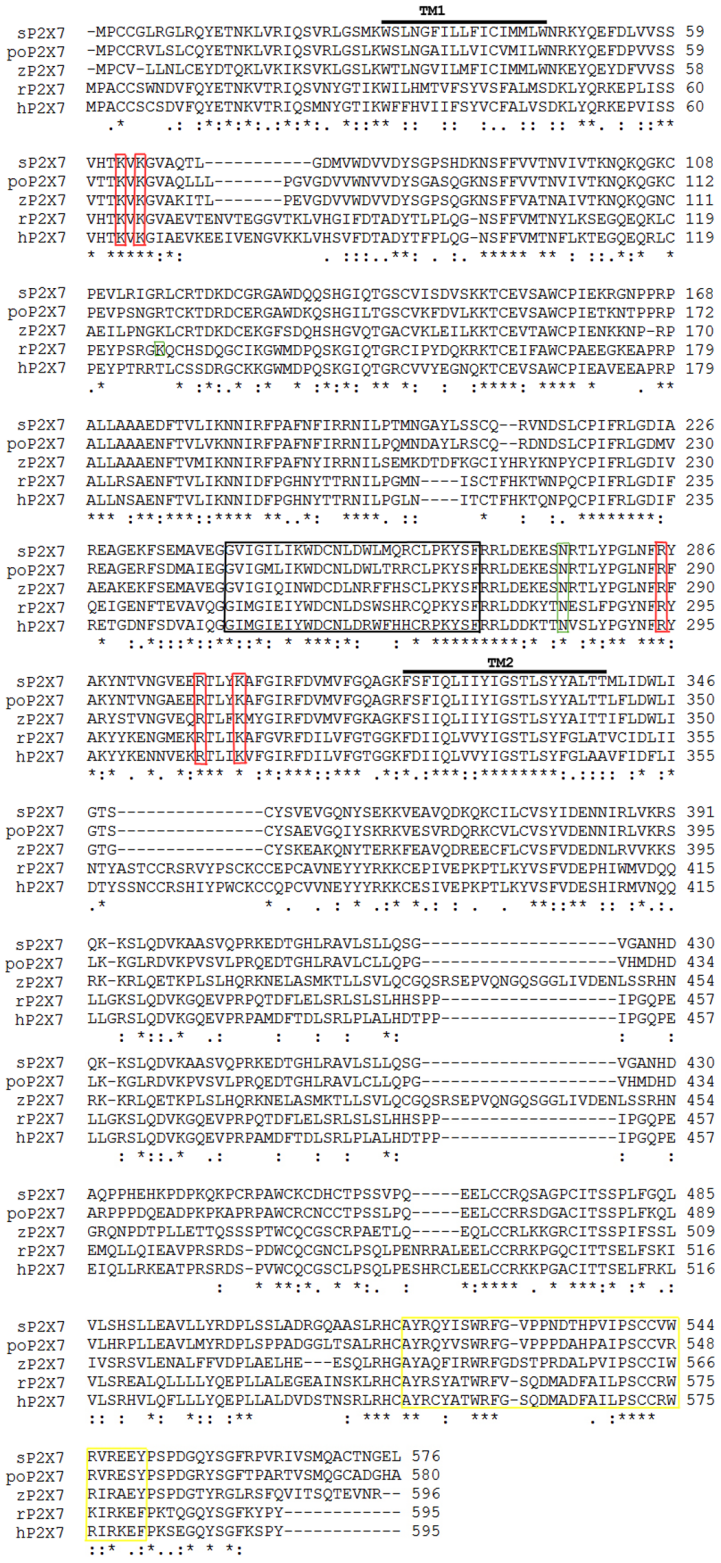
Multiple sequence alignment of poP2X7 receptor with selected P2X7 receptor proteins. Sequence alignment was carried out by ClustalW program. Representative P2X7 receptor proteins from different species with GenBank accession numbers are hP2X7 (*Homo sapiens*, NP_002553), rP2X7 (*Rattus norvegicus*, NP_062129), sP2X7 (*Sparus aurata*, CAI59608), zP2X7 (*Danio rerio*, AAI63071) and poP2X7 (*Paralichthys olivaceus*, KC748421). Highly conserved (:), less conserved (.) and identical (*) amino acid residues identified in all the proteins are indicated. TM: transmembrane domain. The P2X family signature motif (^244^Gly–^270^Phe) was boxed in black. The two residues ^127^Lys and ^284^Asn accounted for species difference of P2X7 receptors in ATP/BzATP agonist sensitivity were boxed in green [28]. Five important residues for nucleotide binding were boxed in red and the predicted LPS/lipid-binding domain was boxed in yellow.

**Figure 2:**
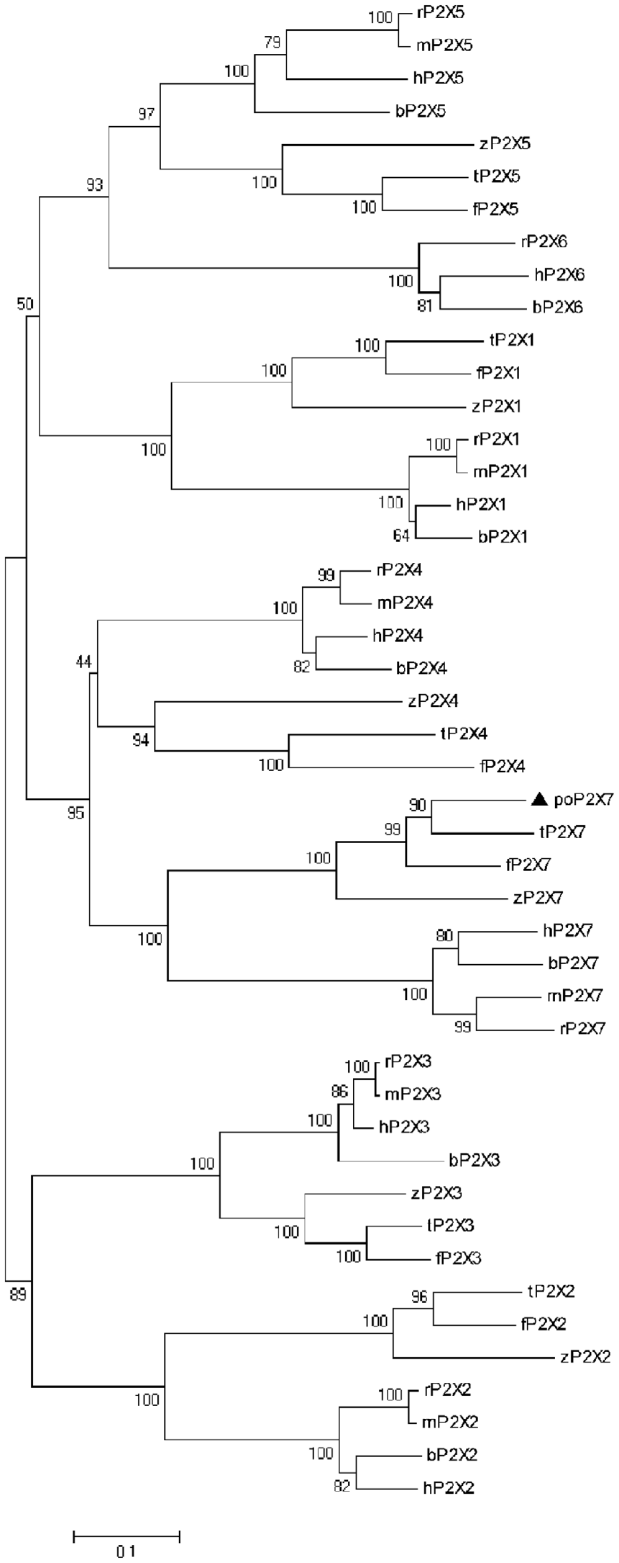
Phylogenetic relationship of Japanese flounder poP2X7 with selected vertebrate P2X receptor family proteins. Maximum-likelihood phylogenetic tree was generated using MEGA 5.1 program. The bar indicates the distance and the number at each node indicates the percentage of bootstrapping after 1000 replications. The GenBank accession numbers of selected P2X receptor proteins are hP2X1 (AAC24494.1), rP2X1 (NP_037129.1), mP2X1 (NP_032797.3), bP2X1 (NP_001192729.1), zP2X1 (NP_945333.1), tP2X1 (XP_003458088.1), fP2X1 (XP_003977018.1), hP2X2 (NP_733782.1), rP2X2 (NP_446108.2), mP2X2 (AAK95327.2), bP2X2 (NP_001179572.1), zP2X2 (NP_945334.1), tP2X2 (XP_003451709.1), fP2X2 (XP_003974964.1), hP2X3 (NP_002550.2), rP2X3 (NP_112337.2), mP2X3 (NP_663501.2), bP2X3 (XP_608941.3), zP2X3 (NP_945337.2), tP2X3 (XP_003456590.1), fP2X3 (XP_003972130.1), hP2X4 (NP_001243725.1), rP2X4 (NP_113782.1), mP2X4 (NP_035156.2), bP2X4 (NP_001029221.1), zP2X4 (NP_705939.1), tP2X4 (XP_003448602.1), fP2X4 (XP_003974770.1), hP2X5 (NP_002552.2), rP2X5 (NP_542958.2), mP2X5 (NP_201578.2), bP2X5 (XP_005195684.1), zP2X5 (NP_919394.1), fP2X5 (XP_003976410.1), tP2X5 (XP_003456207.1), hP2X6 (AAF13303.1), rP2X6 (CAA66044.1), bP2X6 (XP_005195206.1), hP2X7 (NP_002553), rP2X7 (NP_062129), bP2X7 (NP_001193445), mP2X7 (CAD33539), zP2X7 (NP_945335), fP2X7 (XP_003974725), tP2X7 (XP_003444500.1) and poP2X7 (KC748421). h, Human (*Homo sapiens*); r, rat (*Rattus norvegicus*); m, mouse (*Mus musculus*); b, cattle (*Bos Taurus*); z, zebrafish (*Danio rerio*); f, fugu (*Takifugu rubripes*); t, Nile tilapia (*Oreochromis niloticus*); po, Japanese flounder (*Paralichthys olivaceus*).

The deduced poP2RX7 protein is comprised of 580 amino acids with an estimated molecular mass of 65.0 kDa and an isoelectric point of 9.18. In general, poP2RX7 shows about 60–88% sequence similarity and 45–78% sequence identity with known P2RX7 proteins and shares higher sequence similarity with fish P2RX7 members. Particularly, among the fish P2RX7 proteins, poP2RX7 shares the highest sequence identity (78%) with seabream (*Sparus aurata L.*) P2RX7 [19]. Multiple alignment of selected fish and mammalian P2RX7 protein sequences clearly reveals that poP2RX7 possesses several conserved residues including 5 important residues for nucleotide binding and 10 cysteine residues that form five disulphide bonds in the extracellular loop (Fig. 1). Compared with mammalian P2RX7s, however, poP2RX7 bears only a partial cysteine-rich 18-amino-acid segment in the distal juxtamembrane region, which contributes to specific P2RX7 properties and involves the regulation of pore dilatation [26]. Protein domain analysis reveals that in addition to the P2X receptors signature motif (^244^Gly–^270^Phe) and cysteine-rich domain, poP2RX7 also bears several other important domains including a ZASP-like motif (^548^Arg–^573^Gln), 13 protein kinase C phosphorylation sites, and 3 Casein kinase II phosphorylation sites. Glycosylation status has been evidenced to be critical for human [27], rat and mouse [28] P2RX7s trafficking and function. In poP2RX7, three putative N-linked glycosylation sites with the consensus sequence Asn-X-Ser/Thr (at amino acid positions 180, 217 and 279, respectively) were found by NetNGlyc program. Further phylogenetic analysis placed poP2RX7 and other P2RX7 proteins within their own cluster apart from other P2RX members (Fig. 2), confirming that poP2RX7 represents a new member of P2RX7 protein family.

### Tissue distribution of *poP2RX7* mRNA transcript

**Figure 3:**
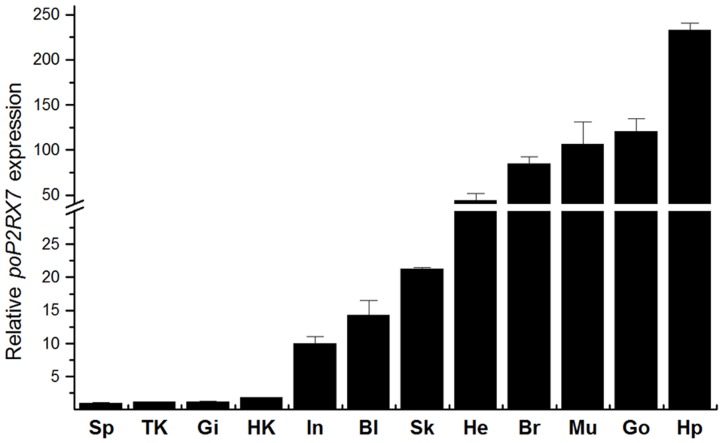
Tissue distribution of Japanese flounder *poP2RX7* mRNA. *poP2RX7* mRNA expression in healthy *P. olivaceus* tissues was analyzed by qRT-PCR with *β-actin* as an internal reference gene. Bl: blood; Br: brain; Gi: gill; HK: head kidney; TK: trunk kidney; He: heart; Hp: hepatopancreas; Sk: skin; Go: gonad; Sp: spleen; In: intestine. The identities of all PCR products were confirmed by DNA sequencing. Values are presented as means ± standard deviation from triplicate experiments.

The presence of *poP2RX7* mRNA transcript in unstimulated healthy adult *P. olivaceus* tissues were examined by qRT-PCR analysis. As shown in Fig. 3, *poP2RX7* mRNA was detected in all examined tissues with the highest expression in hepatopancreas, intermediate expression in gonad, muscle, brain, heart, skin, blood and intestine, and less expression in head kidney, gill, trunk kidney and spleen. No PCR products were detected from controls containing all components except reverse transcriptase, ruling out the possibility of genomic DNA contamination (data not shown).

### Pharmacological properties of poP2X7 receptor expressed in *Xenopus* oocytes

**Figure 4:**
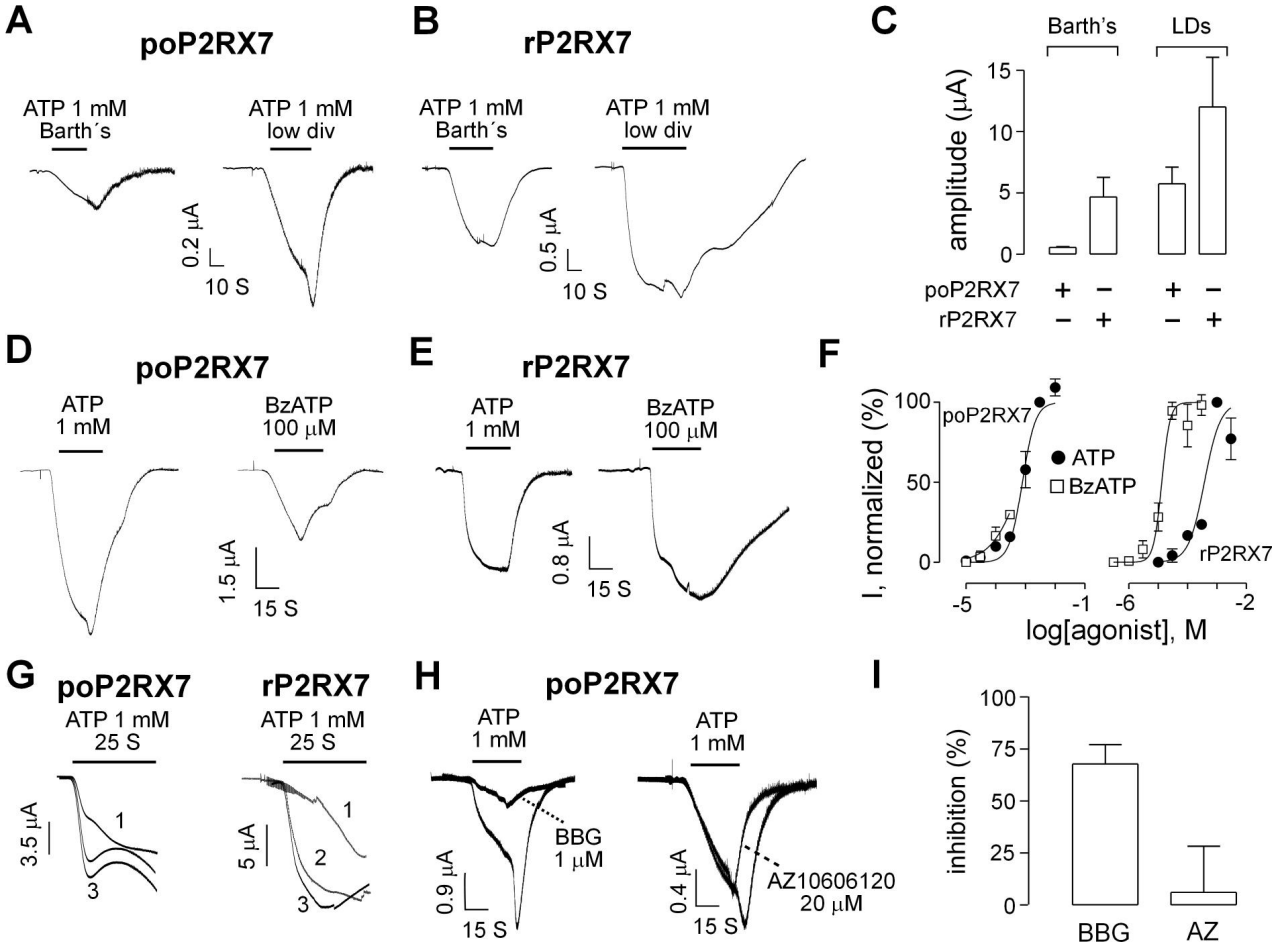
Electrophysiological properties of poP2RX7 expressed in *Xenopus* oocytes. A and B. Representative recordings of individual oocytes expressing poP2RX7 (A) or rP2RX7 (B), currents were gated with 1 mM ATP dissolved in Barth's or low-divalent (LD) media. (C). Summary of the peak amplitudes obtained in Barth's or LD media in oocytes expressing poP2RX7 or rP2RX7. D and E. Currents evoked by 1 mM ATP and 100 µM BzATP from individual oocytes expressing poP2RX7 (D) or rP2RX7 (E), in LD media. (F). Concentration-response curves for BzATP (open squares) and ATP (black circles) for poP2RX7 (left graph) or rP2RX7 (right graph). (G). Current facilitation of poP2RX7 (left recordings) and rP2RX7 (right recordings). In each case, three consecutive ATP pulses were applied to the same oocyte. (H). Representative recording of poP2RX7-expressing oocytes showing the currents evoked by ATP alone and the inhibition induced by pre-application with BBG for 2 min followed by an co-application with 1 µM BBG (left recordings) or 20 µM AZ10606120 (right recordings). (I). Summary of the inhibition induced by BBG or AZ10606120 (AZ) at poP2RX7 (open bars) and rP2RX7 (black bars). *p<0.05, compared with rP2RX7 by Mann-Whitney test, n = 3–5.

To characterize this new receptor, we expressed poP2RX7 protein in *Xenopus* oocytes and compared its properties with the well-studied rat counterpart (rP2RX7). We first tested if oocytes injected with the fish *poP2RX7* were able to induce cationic currents in response to ATP administration. We found that poP2RX7 channel was fully functional as 1 mM ATP induced robust currents (Fig. 4A) with a mean amplitude of 8.6±2.2 µA (n = 26, Fig. 4A and C) when recorded in a Low-divalent (LD) media, containing 0.5 mM Ca^2+^ and 0.1 mM Mg^2+^. Similar to rP2RX7, the presence of divalent cations inhibited the ATP-evoked currents of poP2RX7 (Fig. 4A and B), and the peak amplitude was reduced by 10-fold in oocytes bathed in Barth's media (containing 1.2 mM Ca^2+^ and 0.8 mM Mg^2+^, Fig. 4A–C).

BzATP is commonly used as a specific agonist for P2RX7-mediated responses due to its higher potency compared to ATP. However, in poP2RX7s, BzATP showed a similar potency to that obtained with ATP with estimated EC_50_s of 743±299 and 790±81 µM for BzATP and ATP, respectively (n = 4–6, Fig. 4F). In contrast, BzATP showed higher affinity than ATP in rat P2RX7, with estimated EC_50_s of 13.3±1.6 and 376±120 µM for BzATP and ATP, respectively (n = 3–4, Fig. 4F). Like its rat counterpart, poP2RX7 also showed current facilitation, a phenomenon that is related to pore dilation (Fig. 4G). We also tested the action of two commonly used P2RX7 antagonists BBG and AZ10606120 and found that the ATP-evoked currents at poP2RX7 could be inhibited by both of the antagonists (Fig. 4H), although AZ10606120 was less potent in poP2RX7 than in rP2RX7 (Fig. 4I).

**Figure 5:**
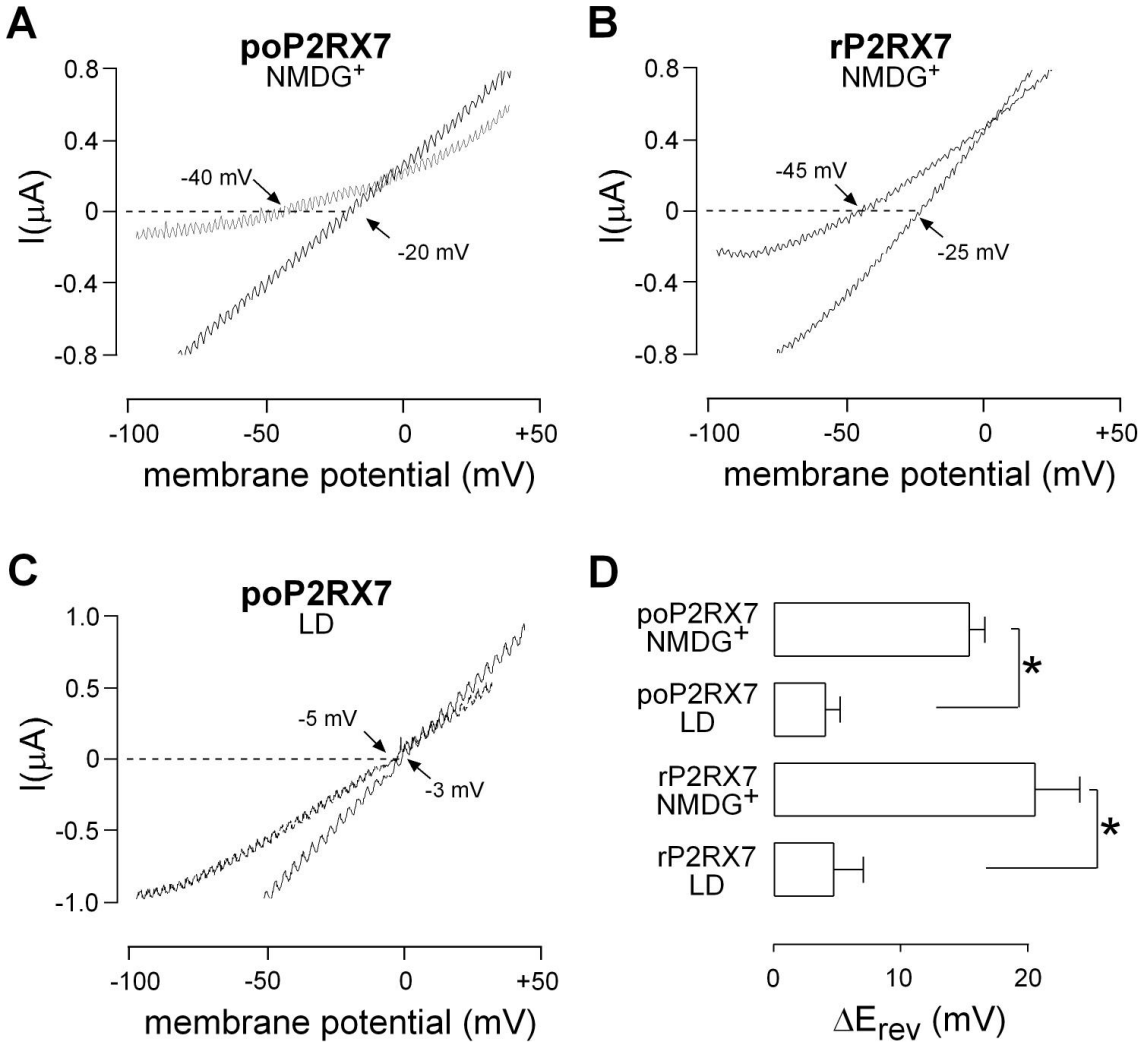
Characterization of poP2RX7 permeability to NMDG^+^. A. Representative −100/+40 mV ramp experiment was applied to an oocyte expressing the poP2RX7 and bathed with NMDG^+^ media. For clarity only two ramps are shown, corresponding to the beginning and after 3 min of continuous application of 1 mM ATP. The values of reversal potential are shown by arrows. B. The same protocol was applied to an oocyte expressing the rP2RX7 and bathed with NMDG^+^ media. C. Representative ramp protocol applied to an oocyte expressing the poP2RX7 and bathed with LD media, no change in reversal potential is observed under these conditions. D. Summary of the changes in reversal potential (ΔE_rev_) after 3 min of ATP application in oocytes expressing the poP2RX7 or the rP2RX7 bathed in NMDG^+^ or LD media. *p<0.05, estimated by Mann-Whitney test. n = 3–7.

Next we studied if the poP2RX7 exhibits changes in its ion permeability during prolonged ATP applications, a phenomenon that probably reflects pore dilation of the functional channel. To accomplish that we performed I-V protocols in oocytes bathed with a NMDG^+^ media, delivering −100/+40 mV ramps protocols (duration of each ramp: 1 S) before and during a 3 min ATP application. Under these conditions we observed a shift in the reversal potential from −28±4 to −13±4 mV in oocytes expressing the poP2RX7 (n = 7, representative experiment in Fig 5A). Similarly we also observed shifts in the reversal potential in oocytes expressing the rP2RX7, from −38±4 to −19±4 mV (n = 6, representative experiment in Fig 5B). However, there is no significant difference between the ΔE_rev_ values of the two P2RXs (poP2RX7 *vs.* rP2RX7). When ramp protocols were applied in oocytes bathed in LD media no significant changes in reversal potential were observed and as expected ΔE_rev_ under these conditions were significant different from oocytes bathed in NMDG^+^ media (Fig. 5C). Finally, both *P. olivaceus* and rat P2RX7 were able to develop NMDG^+^-driven currents in oocytes clamped at −70 mV (Fig. S2). Similar to previous studies [2], [26], the inward NMDG^+^ mediated currents were not deactivated or deactivated very slowly after ATP removal and it was necessary to switch to a sodium-containing media to complete deactivation (Fig. S2C).

### LPS and Poly(I:C)-induced gene expression of *poP2RX7 in vitro*

**Figure 6:**
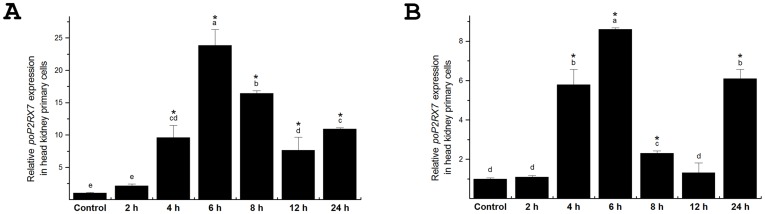
LPS and Poly(I:C)-induced gene expression of *poP2RX7* in *P. olivaceus* head kidney primary cells. *P. olivaceus* head kidney primary cells were prepared as described in *Material and methods* and stimulated with 25 µg/ml (final concentration) LPS (A) or Poly(I:C) (B). Total RNA from different time points (0, 2, 4, 6, 8, 12 and 24 h post stimulation) was extracted and the gene expression changes of *poP2RX7* were determined by qRT-PCR. *β-actin* was employed as an internal reference gene. Values labeled with different *lowercase* letters indicate significant difference (*p*<0.05) among treatments. Asterisks (*) mark the significant up-regulation of *poP2RX7* mRNA compared with the untreated control group (*p*<0.05). Data in this and following figures are the mean ± standard deviation of triplicate determinations from one representative experiment; similar results were obtained on two other separate experiments.

Gram negative bacterial endotoxin lipopolysaccharide (LPS) is a prominent and well-studied pathogen-associated molecular pattern (PAMP) and Poly(I:C), a synthetic analog of double-stranded RNA, is another typical PAMP to mimic viral infection. Head kidney is the principal immune tissue with key regulatory functions in fish. *P. olivaceus* head kidney primary cells were therefore selected as a cell model to examine the gene expression changes of *poP2RX7* in response to LPS and Poly(I:C) stimulations. As shown in Fig. 6A, *poP2RX7* mRNA expression was rapidly induced at 2 h and the peak expression appeared at 6 h post LPS stimulation with expression level about 24 times higher than control group. Notably, this up-regulated expression trend lasted till the end of experiment. When head kidney cells were treated with Poly(I:C), *poP2RX7* gene expression was also significantly up-regulated at 4 h post administration and occurred in a biphasic pattern. The early peak expression appears at 6 h post Poly(I:C) treatment with expression level about 8.6 times higher than control group and the later peak expression appears at 24 h after Poly(I:C) treatment with expression level about 6 times higher than control group (Fig. 6B). These findings indicate that *poP2RX7* is an immune response gene which is highly inducible by different PAMPs challenges.

### *poP2RX7* gene expression in response to bacterial infections *in vivo*

**Figure 7:**
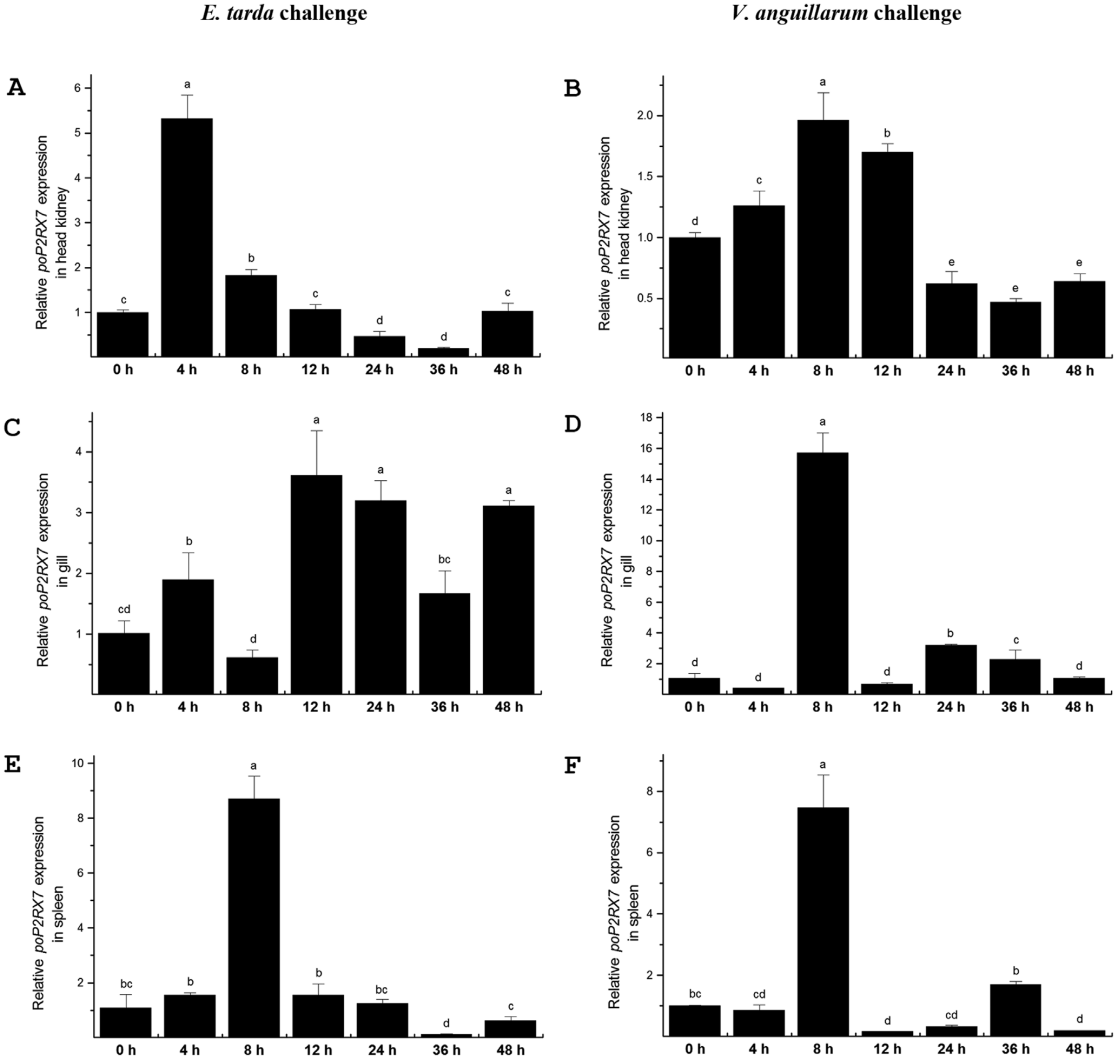
Temporal expression analysis of *poP2RX7* mRNA transcripts in response to bacterial infections by real-time PCR. Animals (average 20±3 g) were injected intraperitoneally with *E. tarda* or *V. anguillarum* and sacrificed at 0, 4, 8, 12, 24, 36 and 48 h after injection. Head kidney, spleen and gill tissues from five individual animals were dissected, collected at each time point and total RNA of each tissue was pooled. The expression changes of *poP2RX7* mRNA in head kidney (A and B), gill (C and D) and spleen (E and F) upon *E. tarda* (A, C and E) and *V. anguillarum* (B, D and F) infections are relative to *poP2RX7* gene expression in control experiment of each time point (normalized to 1). *β-actin* was employed as an internal reference gene. Values marked with different *lowercase* letters indicate significant difference (*p*<0.05) among treatments.

Bacteria *E. tarda* and *V. anguillarum* are severe disease causing pathogens and their outbreak may result in 50–80% mortality and huge economic losses in Japanese flounder *P. olivaceus* mariculture industry in China. Next, we examined *poP2RX7* gene expression changes in response to the infections by the two bacterial pathogens in representative fish immune-related tissues. In head kidney, *poP2RX7* mRNA was rapidly and significantly up-regulated at 4 h post *E. tarda* injection with expression level about 5.3 times higher than control group and then returned to basal level at 12 h post infection (Fig. 7A); when Japanese flounder was infected with *V. anguillarum*, the expression of *poP2RX7* mRNA transcripts increased up to 2 times relative to the control group at 8 h post infection, and dropped to less than the control level at 24 h post infection (Fig. 7B). In gills, *poP2RX7* gene was rapidly up-regulated at 4 h followed by a surging expression at 12 h post *E. tarda* infection and this sustained high expression level lasted till the end of experiment (Fig. 7C); the expression of *poP2RX7* mRNA transcript in gill was also substantially up-regulated to a peak level at 8 h post *V. anguillarum* infection with expression level about 15 times higher than control group (Fig. 7D). In spleen, the expression of *poP2RX7* gene was significantly up-regulated at 8 h post *E. tarda* challenge with expression level about 8.7 times higher than control group and then declined quickly to the basal level (Fig. 7E); The *poP2RX7* gene expression in spleen was also rapidly induced at 8 h post *V. anguillarum* challenge with expression level about 7.4 times higher than control group followed by a second peak expression at 36 h (Fig. 7F). The significant and rapid up-regulation of *poP2RX7* mRNA in response to the bacterial infections *in vivo* further indicates that *poP2RX7* is an essential gene engaged in Japanese flounder innate immune response.

### Activation of poP2RX7 channel up-regulates *IL-1β* and *IL-6* gene expression

**Figure 8:**
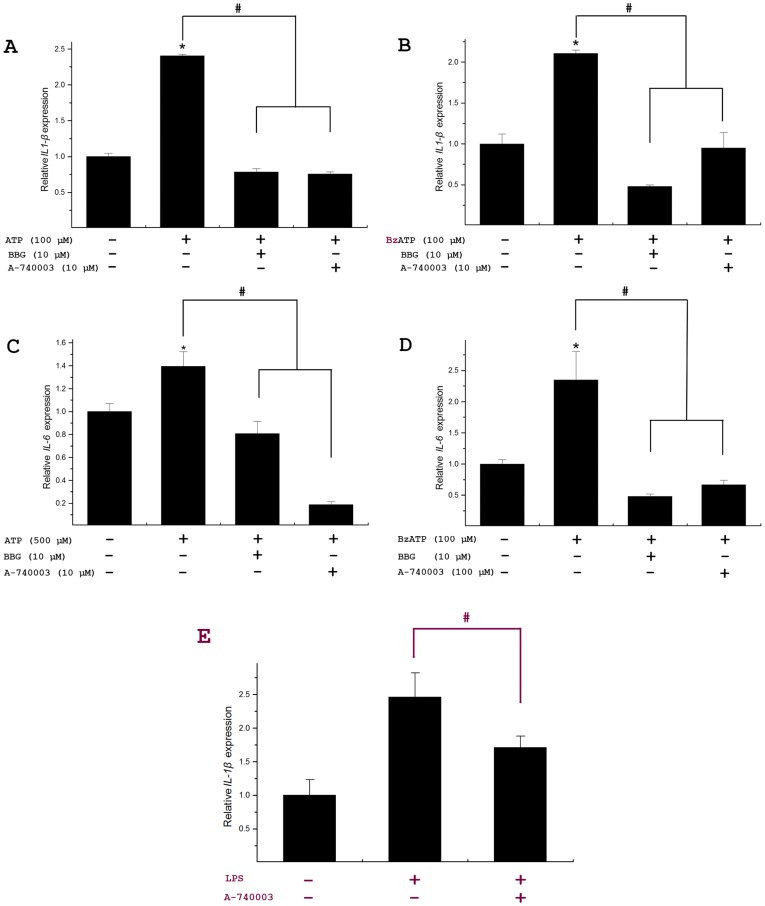
PoP2RX7-mediated gene expression of *IL-1β* and *IL-6* in Japanese flounder head kidney primary cells. (A–D) The involvement of poP2RX7 in ATP-evoked *IL-1β* and *IL-6* expression. Japanese flounder head kidney primary cells (1.0×10^7^/well) were pre-incubated with or without selective P2RX7 inhibitors BBG or A-740003 for 2 h and then co-treated with 100, 500 µM ATP or 100 µM BzATP for 30 min to active poP2RX7. The cells were finally incubated with normal culture medium for 2 h and used for RNA isolation. (E) The involvement of poP2RX7 in LPS-induced *IL-1β* gene expression. Japanese flounder head kidney primary cells were pre-treated with or without P2RX7 antagonist A-740003 (final concentration, 10 µM) for 2 h and then primed with 20 µg/ml LPS in the presence or absence of the P2X7R antagonist for 3 h. The gene expression changes of *IL-1β* (A, B and E) and *IL-6* (C, D) were determined by qRT-PCR with *β-actin* as an internal reference gene. Asterisks (*) mark the significant difference between experimental and untreated control groups (normalized to 1), *p*<0.05. ^#^, values differ at *p*<0.05.

The involvement of P2RX7 in cytokine expression and release has been studied in rat microglia [29]. To test whether poP2RX7 remains the conserved role in fish, we examined the influence of poP2RX7 channel activation on the gene expression of predominant pro-inflammatory cytokines *IL-1β* and *IL-6* in *P. olivaceus* head kidney primary cells by qRT-PCR. As shown in Fig. 8A-D, the expression of *IL-1β* and *IL-6* gene was significantly up-regulated when poP2RX7 was activated by treatment Japanese flounder head kidney primary cells 30 min with 100 and 500 µM ATP or 100 µM (optimized final concentration) synthetic ATP derivative and P2RX7 agonist BzATP. This up-regulated expression could be inhibited by P2RX7 antagonist BBG or A-740003, suggesting that the elevated gene expression for *IL-1β* and *IL-6* might be induced by poP2RX7 activation. However, we can not exclude the possibility that other P2 receptor members may also contribute the ATP-evoked *IL-1β* and *IL-6* expression and we do not know why the commonly used P2RX7 antagonists (BBG and A-740003) in some cases can inhibit the ATP/BzATP-evoked cytokine gene expression to less than control level. We further examined the involvement of poP2RX7 in LPS-induced *IL-1β* gene expression. As shown in Fig. 8E, the LPS-induced *IL-1β gene* expression was significantly inhibited by P2RX7 antagonist A-740003, suggesting that poP2RX7 is not only involved in ATP-evoked but also in LPS-induced *IL-1β* gene expression.

## Discussion

P2RX7 protein is structurally distinguished from other subtypes of P2RX family by its long intracellular C-terminal tail and distinctive channel properties and has received much more attention than other P2RXs because of its important roles in inflammation and innate immunity in mammals. In fish, however, the properties and biological functions of P2RX7 are still very limited. Given the economic importance and species diversity, finding more details about P2RX7 in fish is particularly interesting and important to understand the receptor's properties and potential immune functions from an evolutionary point of view. Here, we identified and characterized a novel member of fish *P2RX7* cDNAs in Japanese flounder, termed *poP2RX7*, and investigated its involvement in fish immune response.

Comparative analyses of the primary structure of poP2RX7 protein with known vertebrate counterparts reveal that strikingly conserved structures (i.e. two transmembrane domains, the long C terminus, the P2X family signature motif, five important residues for nucleotide binding and the LPS/lipid-binding domain) have been kept throughout evolution. In addition, poP2RX7 protein has three putative glycosylation sites that have been demonstrated to be critical for P2RX7s trafficking, function and ATP potency in mammals [27], [28]. Phylogenetic analysis revealed that poP2RX7 was located into the P2RX7s cluster which is distinct from the clusters of other P2RX members, demonstrating that poP2RX7 represents a new member of P2RX7 family.

In healthy Japanese flounder, *poP2RX7* mRNA was detected in all tested tissues with dominant expression in hepatopancreas and relative higher expression in gonad, muscle, brain, heart, skin, blood and intestine. In immune-related tissues including head kidney, gill and spleen, however, *poP2RX7* mRNA expression is lower. The relatively lower mRNA expression of *P2RX7* was also observed in the lymphoid organs (head kidney and spleen) of gilthead seabream *S. aurata* (another kind of marine fish), but the higher expression of *P2RX7* mRNA was found in head-kidney macrophages and acidophilic granulocytes, the two professional phagocytic cells of the seabream [19]. In addition, as head kidney and spleen are comprised by several cell types and immune cells may only represent a small population of the mixed cells, the lower expression of *poP2RX7* in these tissues therefore may not imply its lower expression in specific immune cell types.

In further experiments, we characterized the channel properties of poP2RX7 expressed in *Xenopus* oocytes by electrophysiological recordings. Our results demonstrated that poP2RX7 is a functional channel with properties that are common to other P2RX7 homologues in general. For example, divalent cations such as calcium and magnesium can inhibit the activity of mammalian P2RX7 by altering the affinity of ATP binding to the receptor in an allosteric manner [30], [31] and this character was preserved in poP2RX7. Another similarity between poP2RX7 and rP2RX7 is their ability to increase its permeability to large organic cations during prolonged activation, a phenomenon related to the dilation of the channel pore. This result is somehow surprising taking into account that the poP2RX7 lacks the C-terminal cysteine-rich region that has been proposed to be important for pore dilation by some investigators [26]. However, other authors have found that removal of this region increases rather than decreases its permeability to NMDG^+^ [2]. Three observations of the present work allow us to conclude that the poP2RX7 pore dilates: these are current facilitation (Fig. 4G), shift in reversal potential (Fig. 5) and NMDG^+^-driven currents (Fig. S2). These results suggest that although the C-terminal cysteine-rich region may have an important regulatory role, there must be other determinants in the P2RX7 that are critical for the development and maintenance of pore dilation. Although a first study suggested that pore dilation is not observed when the P2RX7 is expressed in *Xenopus* oocytes [32], later studies have found this phenomenon in oocytes expressing the P2RX7 [33], P2RX2 [34] and P2RX4 [35], suggesting that pore dilation is a characteristic feature of P2X receptors.

However, in contrast to rat, human and other known fish P2RX7s, poP2RX7 shows similar agonist potency for ATP and BzATP. This distinct feature makes poP2RX7 an attractive model to study purinergic receptor structure-activity relationship. Moreover, poP2RX7 showed to be less sensitive to some P2RX7 antagonists such as AZ10606120, and the analysis of the differences in receptor sequences could help to understand the mechanisms of these antagonists. Important differences in antagonist effectiveness have been reported between the P2RX7s of different species (human *versus* rat [36] and mammalian *versus* nonmammalian [19]). A previous study has also demonstrated that P2RX7 agonist potency is determined solely by its ectodomain and the residues ^127^Lys and ^284^Asn are accounted for species difference (rat *versus* mouse) of P2RX7s in ATP/BzATP agonist sensitivity [28]. Sequence alignment revealed that ^127^Lys was replaced with ^120^Arg but the glycosylation site ^284^Asn was conserved in poP2RX7. Whether ^284^Asn is glycosylated and ^120^Arg replacement in poP2RX7 is involved in the distinct agonist sensitivity need to be addressed in the future.

It has been reported that LPS can stimulate the synthesis of *P2RX7* [37]. In the present study, we demonstrated that *poP2RX7* mRNA expression was rapidly and significantly up-regulated in response to LPS stimulation *in vitro*. In addition, P2RX7 has been demonstrated to play an important role in regulating inflammatory responses during acute viral infection in mouse [38]. In our experiments, *poP2RX7* mRNA transcript was also substantially up-regulated by immune challenge with Poly(I:C), which is a synthetic analog of double-stranded RNA to mimic viral infection. Taken together, these experiments indicate that *poP2RX7* may serve as an important innate immune response gene during the early stage of bacterial and viral infections in fish.

P2RX7 comprises an important part of the host arsenal against invading pathogens [39]. Next, we selected spleen, gill and head kidney as representative immune-related tissues to examine the *in vivo* gene expression profile of *poP2RX7* in response to bacteria *E. tarda* and *V. anguillarum* infections. Head kidney is a major piscine immune tissue, spleen is another putative major innate immune tissue in fish, and gill is a multifunctional immune involved organ which is directly subjected to expose in the aquatic environment containing a huge microbial biomass [40]. In healthy Japanese flounder, *poP2RX7* mRNA expresses lower in these tissues. However, the expression of *poP2RX7* mRNA was significantly up-regulated upon the infections by both of the bacteria. Notably, among the three tested immune-related tissues *poP2RX7* gene expression was rapidly induced in gills in response to *E. tarda* infection and the sustained up-regulation lasted till the end of the experiment. It has been demonstrated that P2RX7 expression was significantly augmented in the lungs of mice infected with *Mycobacterium tuberculosis* and activation of P2RX7 could result in significant reduction of *M. tuberculosis*-colony-forming units [41]. In human beings, P2RX7 was implicated in the innate response to obligate intracellular bacteria of the *Chlamydia* genus [42]. In fish *P. altivelis*, *P2RX7* gene was also up-regulated after *Listonella anguillarum* infection and mediated cell death, phagocytosis and bacterial killing [20]. The infection-induced up-regulation of *poP2RX7* in all tested immune-related tissues thus indicates that *poP2XR7* may play a conserved role against bacterial pathogens.

Activation of P2RX7 by extracellular ATP regulates numerous downstream immune responses such as the release of pro-inflammatory mediators, cell proliferation or death [20], [43]. IL-1β and IL-6 are key mediators of host response to infections and powerful pro-inflammatory cytokines involved in a diverse range of inflammatory and infectious conditions. It has been well established that P2RX7 is responsible for ATP-dependent IL-1β release in mammals [44]. Studies also demonstrated that activation of P2RX7 could induce the expression of cytokine *IL-1β* gene in human beings [45]. We therefore examined whether the activation of poP2RX7 could promote the gene expression of selected cytokines in Japanese flounder. We for the first time showed that activation of poP2RX7 by ATP or BzATP, the selective P2RX7 agonist, indeed stimulated *IL-1β* and *IL-6* mRNA expression in Japanese flounder head kidney primary cells, suggesting *poP2RX7* may function as a critical inflammatory regulator in Japanese flounder innate immunity. However, the up-regulated *IL-1β* and *IL-6* expression showing different agonist (ATP) efficacies indicates that the ATP-evoked expression of the two cytokines may be induced differently by co-activation of P2X7 receptor and other P2 receptor(s). In mammals, bacterial endotoxin LPS induces *IL-1β* mRNA expression [46] and P2RX7 is engaged in LPS-induced cytokine gene expression [47]. In the present study, we showed that LPS stimulation also substantially up-regulated *IL-1β* gene expression in the primary Japanese flounder head kidney cells and this up-regulated expression could be significantly attenuated by treatment the head kidney cells with P2RX7 antagonist A-740003, suggesting poP2RX7 also involved in LPS-induced *IL-1β* gene expression.

In conclusion, we identified and characterized a novel fish *P2RX7* homologue which is engaged in Japanese flounder innate immune response, probably through manipulation of pro-inflammatory cytokine genes expression. These findings will facilitate our understandings of the properties and immunological significance of P2RX7 in lower vertebrates.

## Supporting Information

Figure S1 **Nucleotide and deduced amino acid sequence of poP2RX7 from Japanese flounder** ***P. olivaceus*** **.** The stop codon is marked with an asterisk and the possible polyadenylation signal sequence (AATAAA) in the 3′-untranslated region is underlined. This cDNA sequence has been submitted to GenBank database with accession number KC748421.
(TIF)

Figure S2 **Representative tracings of oocytes expressing the poP2RX7 (A) or the rP2RX7 (B) bathed in NMDG^+^ (left recordings) or in LD (right recordings) media.** Voltage was held at −70 mV and 1 mM ATP application is represented by the closed bar. (C) A recording showing the current evoked by 1 mM ATP (closed bar) in an oocyte bathed with NMDG^+^ media and the current increase subsequent current deactivation that was achieved after switching to sodium-containing LD media.
(TIF)
