# Supplementary figures and images for: Molecular Characterization and Expression Analysis of ATP-Gated P2X7 Receptor Involved in Japanese Flounder (Paralichthys olivaceus) Innate Immune Response
Source: PLoS One. 2014 May 5;9(5):e96625. doi: 10.1371/journal.pone.0096625 (PMC4010493; doi:10.1371/journal.pone.0096625)

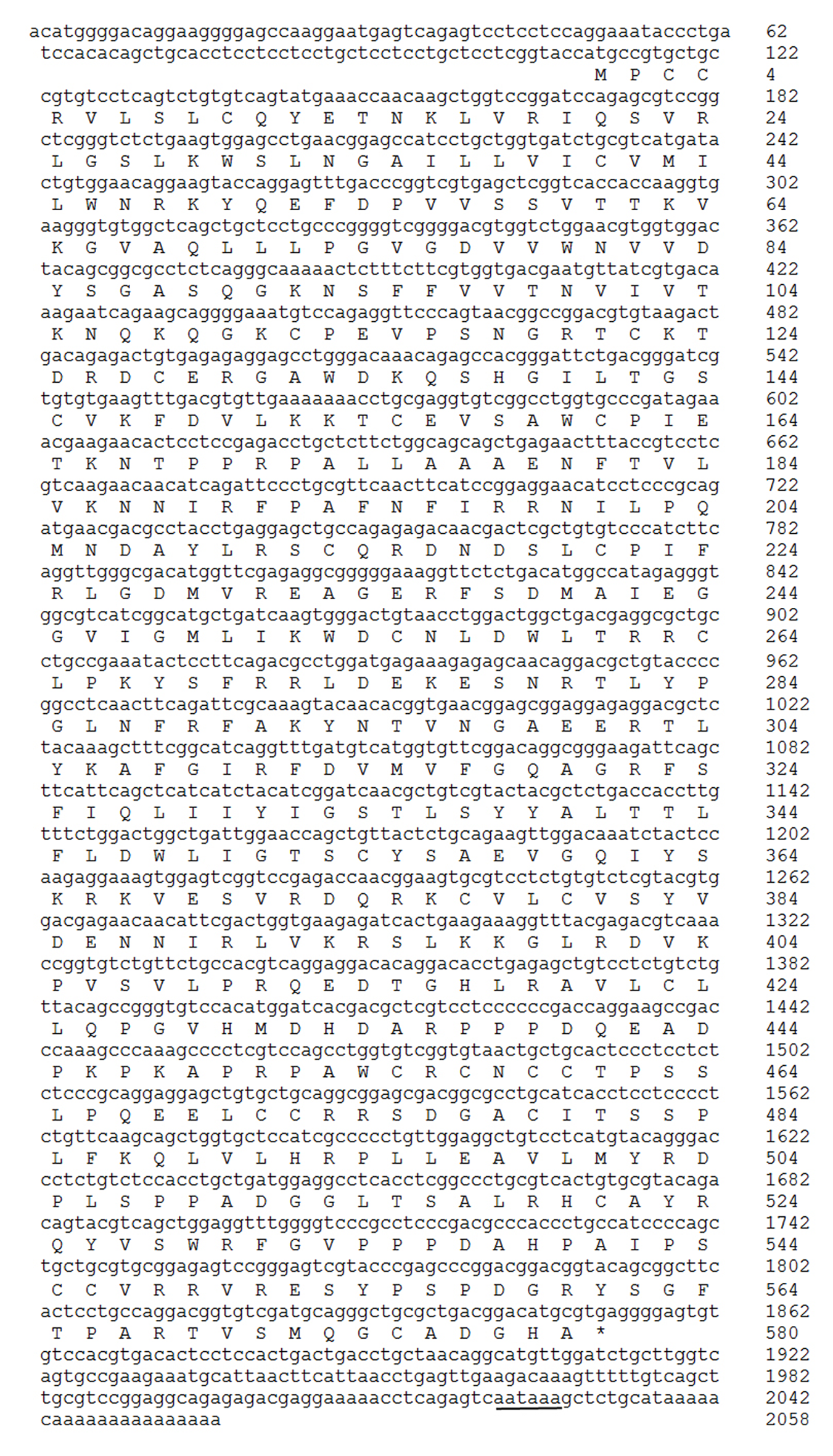

Supplement: Figure S1 — Nucleotide and deduced amino acid sequence of poP2RX7 from Japanese flounder P. olivaceus . The stop codon is marked with an asterisk and the possible polyadenylation signal sequence (AATAAA) in the 3′-untranslated region is underlined. This cDNA sequence has been submitted to GenBank database with accession number KC748421. (TIF) [file pone.0096625.s001.tif]

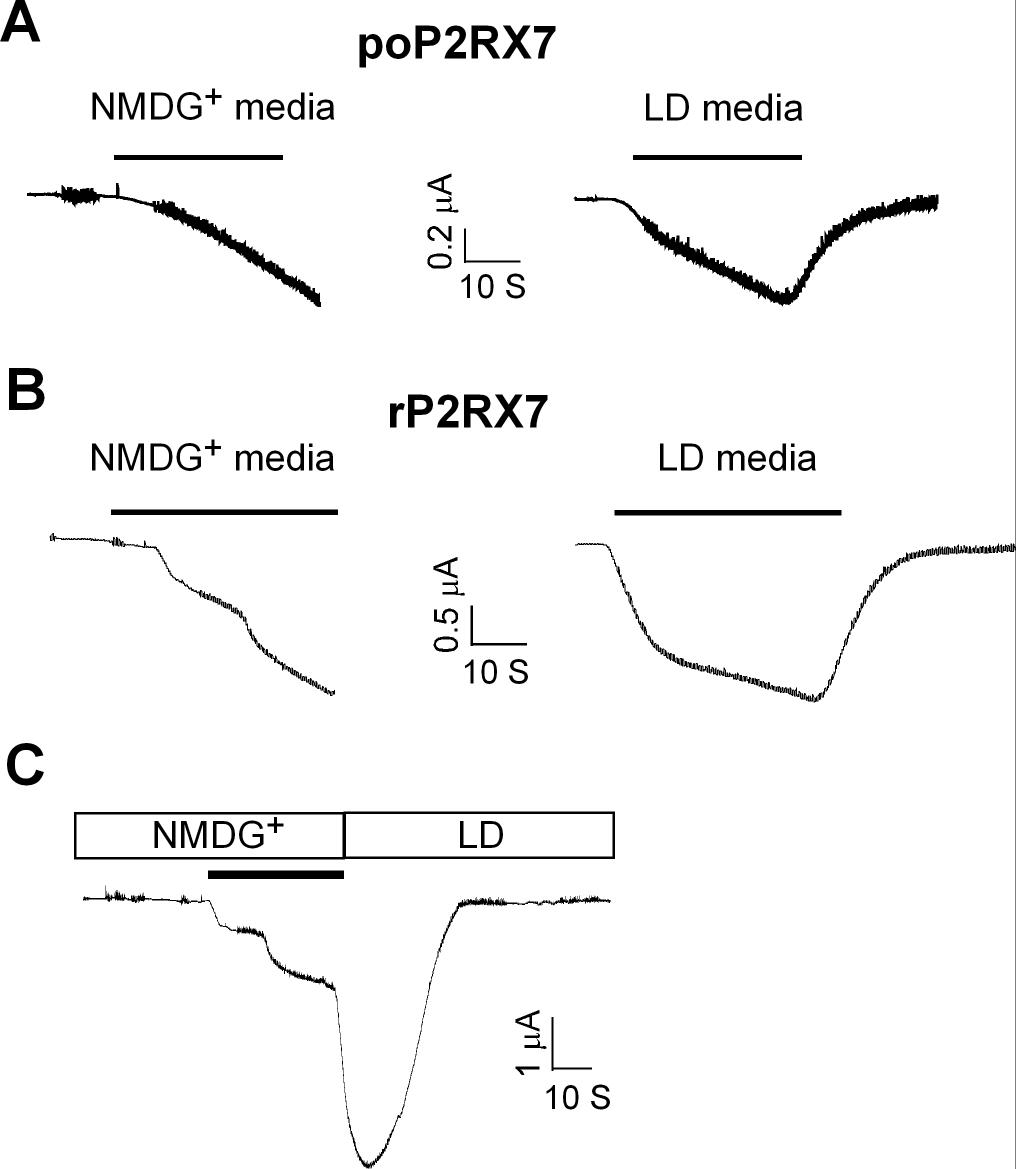

Supplement: Figure S2 — Representative tracings of oocytes expressing the poP2RX7 (A) or the rP2RX7 (B) bathed in NMDG+ (left recordings) or in LD (right recordings) media. Voltage was held at −70 mV and 1 mM ATP application is represented by the closed bar. (C) A recording showing the current evoked by 1 mM ATP (closed bar) in an oocyte bathed with NMDG+ media and the current increase subsequent current deactivation that was achieved after switching to sodium-containing LD media. (TIF) [file pone.0096625.s002.tif]
